# Precise discrimination of mycobacterial pulmonary diseases via multimodal machine learning integrating chest CT and clinical markers

**DOI:** 10.3389/fmed.2026.1858126

**Published:** 2026-07-09

**Authors:** Yangyi Jin, Jindun Ding, Jinsheng Ouyang, Zhiye Yao, Liping Wang, Ruisong Xu, Xuewen Jin

**Affiliations:** 1 Department of General Internal Medicine, Zhejiang Cancer Hospital, Hangzhou, Zhejiang, China; 2 Department of Respiratory and Critical Care Medicine, The People’s Hospital of Yuhuan, Yuhuan, China; 3 Department of Respiratory and Critical Care Medicine, The First Affiliated Hospital of Wenzhou Medical University, Wenzhou, China; 4 Wenzhou Institute, University of Chinese Academy of Sciences, Wenzhou, China; 5 Shanghai Key Lab of Modern Optical System, Terahertz Spectrum and Imaging Technology Cooperative Innovation Center, Terahertz Technology Innovation Research Institute, University of Shanghai for Science and Technology, Shanghai, China

**Keywords:** machine learning, multimodal data integration, nontuberculous mycobacterial lung disease, pulmonary tuberculosis, rapid diagnostic differentiation

## Abstract

**Introduction:**

Differentiating *Mycobacterium tuberculosis lung disease* (MTB-LD) from nontuberculous mycobacterial lung disease (NTM-LD) remains clinically challenging because of overlapping symptoms and imaging features, as well as the slow turnaround of conventional culture-based diagnostics.

**Methods:**

This retrospective study enrolled 102 patients with microbiologically confirmed mycobacterial lung disease, including 53 patients with MTB-LD and 49 patients with NTM-LD. We developed and validated an interpretable multimodal machine-learning framework integrating clinical symptoms, hematological biomarkers, and high-resolution computed tomography (HRCT) features. Three representative classifiers, including k-nearest neighbors, logistic regression, and random forest, were used to evaluate the discriminative contribution of different feature modalities.

**Results:**

Multimodal integration of HRCT, clinical, and laboratory features showed better discriminative performance than single-modality approaches. Among the three representative classifiers, the random forest model achieved the best hold-out test performance, with an AUC of 0.92, sensitivity of 0.89, specificity of 0.93, and F1-score of 0.90. Key predictive contributors included cystic bronchiectasis, tree-in-bud sign, fever, and selected laboratory biomarkers.

**Discussion:**

These findings suggest that routinely available multimodal clinical data may provide preliminary decision support for MTB-LD/NTM-LD differentiation. However, the proposed framework should be regarded as an exploratory decision-support tool, and external validation is required before clinical implementation.

## 1 Introduction

Mycobacterial lung diseases, including *Mycobacterium tuberculosis* lung disease (MTB-LD) and nontuberculous mycobacterial lung disease (NTM-LD), are major infectious pulmonary conditions globally (1). Tuberculosis (TB) remains a critical public health challenge, while the incidence of NTM-LD has been rising, particularly among immunocompromised individuals due to factors such as aging, chronic diseases, and the widespread use of immunosuppressive therapies (2, 3). In China, the increasing rates of NTM isolation further highlight the underreported burden of this disease (4). MTB-LD and NTM-LD share overlapping symptoms such as chronic cough, fever, night sweats, weight loss, and pulmonary damage (5). Chest CT often shows similar features, including cavitation, bronchiectasis, and tree-in-bud opacities (6). However, treatment paradigms diverge fundamentally: MTB-LD responds to standard anti-tuberculosis therapy, whereas NTM-LD often requires prolonged, species-specific antibiotic regimens and may not always necessitate treatment (7). Accurate and timely discrimination between these two entities is therefore critical for appropriate patient management and outcomes.

Conventional diagnostic approaches remain inadequate for this purpose. Acid-fast bacilli smear microscopy cannot differentiate MTB from NTM, and culture-based methods, although the gold standard, are hampered by slow turnaround times (weeks), high cost, and technical demands (8–10). Nucleic acid amplification tests offer improved speed but are not universally available and may yield false positives or negatives (11, 12). Radiological diagnosis, even by experienced clinicians, is subjective and prone to inter-observer variability, often leading to misclassification and inappropriate treatment (13, 14). These limitations underscore the urgent need for rapid, accurate, and accessible diagnostic tools.

Recent advances in artificial intelligence and machine learning have shown promise in medical imaging-based diagnosis of pulmonary infectious diseases, particularly tuberculosis screening and CT-based pulmonary lesion analysis (15–17). Several studies have further applied radiomics, machine learning, or deep learning to chest CT or chest radiography for differentiating MTB-LD from NTM-LD (18–22). Nevertheless, these efforts have predominantly relied on unimodal imaging data, overlooking the complementary value of clinical symptoms, laboratory biomarkers, and immunological indicators that are routinely collected in clinical practice (23). A diagnostic approach that integrates multimodal data, such as demographics, symptoms, hematological and inflammatory markers, and high-resolution CT (HRCT) features, could more comprehensively capture the complex pathophysiology underlying these diseases and improve classification accuracy. Given that several HRCT findings have already been reported to differ between MTB-LD and NTM-LD, this study focused on determining whether established radiological patterns could provide added discriminative value when integrated with routinely available clinical symptoms and laboratory biomarkers.

In this study, we aimed to develop and validate an interpretable multimodal machine-learning framework to evaluate the diagnostic contribution of routinely available HRCT features, clinical symptoms, and laboratory biomarkers for differentiating MTB-LD from NTM-LD. Moreover, we used representative classifiers to examine whether multimodal integration provides more robust discrimination than single-modality data.

## 2 Materials and methods

### 2.1 Study population

This retrospective study enrolled patients diagnosed with mycobacterial lung disease at Yuhuan People’s Hospital, Zhejiang Province, China, between January 2019 and December 2022. The cohort comprised 53 patients with *Mycobacterium tuberculosis* lung disease (MTB-LD) and 49 patients with nontuberculous mycobacterial lung disease (NTM-LD), all confirmed by microbiological evidence. The study protocol received approval from the Institutional Ethics Committee of the People’s Hospital of Yuhuan (Approval No. 2023-012). The research was conducted in strict adherence to relevant guidelines and regulations, and informed consent was obtained from all participants and/or their legal guardians.

Patients were included if they had a pathogen-confirmed diagnosis of either MTB-LD or NTM-LD via sputum culture or molecular diagnostic methods, and if pulmonary lesions were documented by radiological imaging. Exclusion criteria were concurrent infection with both MTB and NTM, co-infection with other confirmed pulmonary pathogens (such as fungi or common bacteria) that could render imaging interpretation indeterminate, or incomplete essential clinical data.

This was an exploratory retrospective machine-learning study. The sample size was determined by all eligible microbiologically confirmed MTB-LD and NTM-LD cases available at the study institution during the predefined study period, rather than by a formal prospective sample-size calculation. After applying these criteria, 102 eligible patients were retained in the final analytic cohort.

### 2.2 Data collection and feature definition

We developed a standardized data extraction form to systematically collect multidimensional information from all enrolled patients. Data categories included demographics (age and sex), clinical symptoms and signs (cough, sputum production, low-grade fever, night sweats, weight loss), and laboratory results including complete blood count, inflammatory markers, and carcinoembryonic antigen (CEA). All laboratory assays followed standardized clinical protocols. Chest high-resolution computed tomography (HRCT) features were also recorded.

Key radiological manifestations were extracted, covering lesion distribution and the presence or characteristics of cavities, bronchiectasis, nodules, tree-in-bud sign, and pleural effusion. To ensure analytical consistency, imaging features were defined according to established radiological criteria. Cystic bronchiectasis referred to clustered, thin-walled, air-filled cystic structures. Cylindrical bronchiectasis was identified by the signet ring sign on axial images (bronchial diameter equal to or greater than the adjacent pulmonary artery) or parallel tram-track lines on longitudinal sections. Tree-in-bud sign meant centrilobular, branching, or clustered micronodular opacities resembling budding twigs. A cavity was an air-filled space within the lung parenchyma; thin-walled cavities had wall thickness of 3 mm or less with smooth regular inner margins and lucent interior, whereas thick-walled cavities had wall thickness greater than 3 mm, irregular walls, and were often surrounded by consolidation or infiltration. Air bronchogram described branching lucencies representing patent airways within areas of consolidation, indicating alveolar filling with exudate. Consolidation was a relatively homogeneous region of increased parenchymal attenuation obscuring vascular margins. Patchy opacities were ill-defined areas of increased attenuation, which could be ground-glass or consolidative in density. A nodule was a discrete rounded opacity of 3 cm or less in diameter, which could be solid, subsolid (part-solid or pure ground-glass), or mixed density. Calcification was defined as focal high-attenuation lesions with sharp margins, which might appear punctate, laminated, nodular, or popcorn-like.

HRCT features were extracted according to predefined radiological criteria from the available retrospective imaging records. Because this was a retrospective study, the dataset contained the final recorded imaging features rather than independent paired reader-level annotations from multiple radiologists. Therefore, formal inter-observer agreement analysis using Cohen’s Kappa could not be validly reconstructed from the existing dataset.

### 2.3 Statistical analysis

Statistical analyses were performed using SPSS version 26.0. Baseline characteristics were compared between the MTB-LD and NTM-LD groups. Categorical variables were presented as counts and percentages. Continuous variables were expressed as mean ± standard deviation after normality assessment using the Shapiro–Wilk test. For normally distributed data with homogeneous variance, independent samples t-tests were used; non-normally distributed data were analyzed with the Mann–Whitney U test. Statistical significance was set at a two-tailed *p* value less than 0.05. Features that showed significant differences in univariate analysis were further examined in multivariate logistic regression to characterize factors associated with disease classification. The univariate and multivariate analyses were used to describe group-level differences and to support clinical interpretation of variables of clinical interest. These analyses were not interpreted as independent evidence of model generalizability.

### 2.4 ML model construction and validation

#### 2.4.1 Data preprocessing

**Figure 1:**
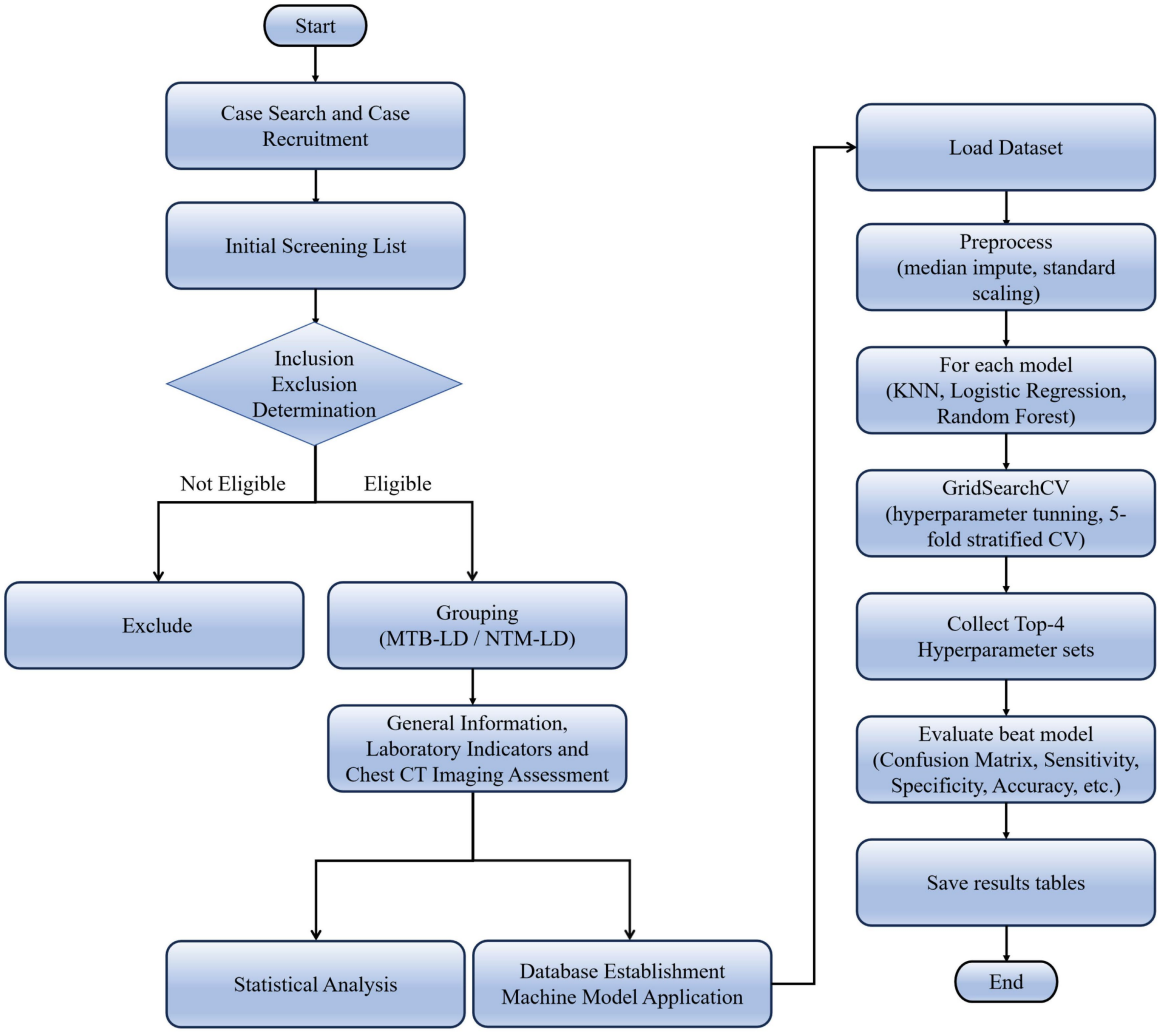
Study workflow diagram.

Classification models were developed using Python 3.10 and the scikit-learn library (version 1.0.2) to differentiate MTB-LD from NTM-LD. The overall modeling workflow, illustrated in Figure 1, consisted of three main stages: data preprocessing, model building with hyperparameter tuning, and performance evaluation with output generation.

We initially collected 28 features covering demographic information, laboratory parameters, and radiological signs. Data preprocessing involved missing value imputation: variables with minimal missing data were imputed by replacing continuous missing values with the median of the respective feature and categorical missing values with the mode (most frequent category). For feature scaling, all continuous features were standardized using Z-score normalization (mean of 0, standard deviation of 1) to enhance model performance and reduce scale-dependent bias. Categorical variables such as sex and location were transformed into sparse binary vectors using one-hot encoding. After preprocessing, we constructed the feature matrix X and target vector y, where MTB-LD cases were labeled as 1 and NTM-LD cases as 0. After the predefined preprocessing procedure, the final feature matrix used for machine-learning model construction contained no missing entries.

This work was revised according to TRIPOD+AI principles for prediction model studies (24). Because this study used manually extracted CT features rather than an end-to-end medical imaging AI system, relevant CLAIM items related to imaging feature definition and interpretation were also considered (Supplementary Table S14) (25). CONSORT-AI was not used because the present study was not a randomized clinical trial evaluating an AI intervention (26).

#### 2.4.2 Model construction and hyperparameter optimization

Three representative classifiers were used not as an exhaustive algorithm benchmark, but as analytical tools to evaluate the discriminative contribution of different feature modalities. KNN, Logistic Regression, and Random Forest were selected to represent distance-based, regularized linear, and nonlinear ensemble learning strategies, respectively. This selection allowed us to examine whether multimodal integration consistently improved MTB-LD/NTM-LD discrimination under different modeling assumptions while maintaining interpretability in a small structured dataset.

The k-nearest neighbors (KNN) model is based on distance metrics. Key hyperparameters tuned included the number of neighbors with values of 3, 5, 7, or 9, and the weight function weights set to either uniform or distance. Logistic regression (LR) is a linear model with L2 regularization to control overfitting. The regularization strength C was tuned among 0.001, 0.1, 1, and 10. We selected the limited-memory Broyden-Fletcher-Goldfarb-Shanno (L-BFGS) algorithm for its balance of convergence efficiency and computational performance. Random forest (RF), originally introduced by Breiman, is an ensemble learning method that can model nonlinear relationships and interactions among predictors (27). Hyperparameters tuned included the number of trees n_estimators (10 or 20), the maximum tree depth max_depth (5, 10, or no constraint), and the minimum number of samples required to split an internal node, min_samples_split (2, 4, or 5).

For hyperparameter tuning, we implemented a comprehensive grid search with GridSearchCV. Hyperparameter combinations were exhaustively evaluated using stratified 5-fold cross-validation to preserve class distribution proportions within each fold. The area under the receiver operating characteristic curve (AUC) served as the primary optimization metric, supplemented by the F1-score to account for potential class imbalance and to balance precision and recall. For each model type, the top four hyperparameter configurations yielding the highest mean cross-validated AUC were retained as candidate models. This top-N approach allowed us to assess model stability and hyperparameter sensitivity. Cross-validation results were used for hyperparameter optimization and candidate-model selection during the model-development stage. Final model comparison was based primarily on hold-out test-set performance rather than tuning-stage cross-validation AUC.

#### 2.4.3 Model evaluation and output

The final selected models (based on cross-validation performance) were evaluated on the hold-out test set. Performance metrics derived from the confusion matrix included:


Sensitivity(Recall):TP/(TP+FN)

Specificity:TN/(TN+FP)

Accuracy:(TP+TN)/(TP+TN+FP+FN)

Area Under the ROC Curve (AUC): Reflecting the model’s overall discriminative ability across all classification thresholds.

Mean F1-scores and AUC values for each model type were calculated and presented in structured tables. We also performed feature importance analysis, such as examining coefficients for LR or using permutation importance or Gini importance for RF, to enhance model interpretability. For hold-out test performance, 95% confidence intervals were estimated for AUC, sensitivity, specificity, accuracy, and F1-score using stratified bootstrap resampling of the hold-out test set. Given the exploratory single-center design and limited sample size, both point estimates and confidence intervals were interpreted cautiously, and model precision requires confirmation in larger external validation cohorts. The finalized models and their evaluation results were saved in structured formats, including serialized model objects and performance reports, for documentation and potential future validation.

#### 2.4.4 Model stability assessment and calibration assessment

To assess potential overfitting and model stability, learning curve analysis was performed for the Random Forest model across three feature modalities: integrated multimodal features, laboratory biomarkers alone, and clinical symptoms alone. Training sample fractions were progressively increased from 20 to 100%, and the corresponding training and validation AUC values were recorded. The training-validation AUC gap was used as an additional indicator of overfitting risk. This analysis was designed to evaluate whether the integrated multimodal feature set provided more stable validation performance than single-modality feature sets.

Calibration analysis was performed for the final integrated-feature Random Forest model. Calibration curves were generated by comparing predicted probabilities with observed outcome proportions across probability bins. Brier score, calibration intercept, calibration slope, and expected calibration error were calculated to evaluate the reliability of predicted probabilities in addition to discrimination performance.

## 3 Results and discussion

### 3.1 Distinct clinical, laboratory, and imaging profiles

The study cohort comprised 102 patients with microbiologically confirmed mycobacterial lung disease (53 MTB-LD, 49 NTM-LD). Demographic analysis (Table 1) revealed no significant difference in age distribution between groups (median 56 vs. 61 years, *p* = 0.678). However, gender composition differed significantly: MTB-LD predominantly affected males (64.2%, 34/53), while NTM-LD showed a female predominance (71.4%, 35/49; *p* < 0.001). Fever was significantly more frequent in MTB-LD (39.6% vs. 2.0%; *p* < 0.001), consistent with the more acute systemic inflammatory response characteristic of tuberculosis. Residential distribution also varied significantly between groups (*p* < 0.001), suggesting possible regional exposure factors influencing infection type (Supplementary Table S1). Other symptoms such as cough, hemoptysis, and dyspnea did not differ significantly, underscoring the overlapping clinical presentation of these two diseases. In addition, laboratory analysis revealed distinct inflammatory and hematological profiles (Supplementary Table S2). The NTM-LD group exhibited significantly higher neutrophil counts (*p* = 0.024) but lower levels of acute-phase reactants including fibrinogen (*p* = 0.001), D-dimer (*p* = 0.010), and procalcitonin (*p* = 0.035). C-reactive protein levels were also lower in NTM-LD, although the difference did not reach statistical significance (*p* = 0.626). These findings suggest that NTM-LD is characterized by a chronic, indolent inflammatory state with a prominent neutrophilic component but a blunted acute-phase response, whereas MTB-LD drives a more robust systemic inflammatory reaction. CEA levels did not differ significantly between the MTB-LD and NTM-LD groups (2.0760 ± 1.4856 vs. 1.5330 ± 0.9107, *p* = 0.087). Therefore, although CEA contributed to model prediction, it should not be interpreted as a significantly different standalone biomarker in univariate analysis.

**Table 1:** Comparison of clinical and laboratory symptoms between the MTB and NTM groups.

Characteristic	Participants with MTB (*n* = 53)	Participants with NTM (*n* = 49)	*p* value
Age (years)	53 (24–84)	49 (34–80)	0.678
Sex			<0.001
Male	34/53	14/49	–
Female	19/53	35/49	–
Fever	21/53	1/49	<0.001
Complete blood count, CBC			
Neutrophils (*10^9^/L)	4.6547 ± 1.7354	5.1122 ± 6.5381	0.024
Inflammatory biomarkers			
Fibrinogen (g/L)	4.6362 ± 1.7189	3.5192 ± 1.1506	0.001
D-dimer (mg/L)	0.9765 ± 1.7020	0.5184 ± 0.6834	0.010
C-reactive protein (mg/L)	34.8188 ± 38.9774	18.0150 ± 36.1604	0.626
Procalcitonin (ng/mL)	0.0929 ± 0.1278	0.0550 ± 0.0444	0.035
Tumor markers			
Carcinoembryonic antigen, CEA (ng/mL)	2.0760 ± 1.4856	1.5330 ± 0.9107	0.087

**Figure 2:**
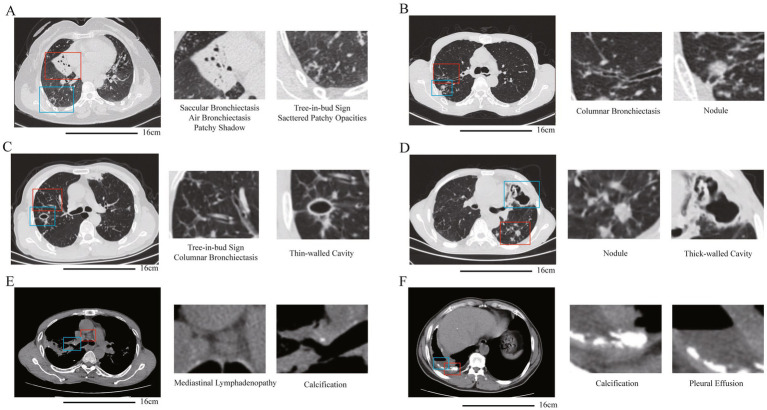
Comparative chest CT imaging features of mycobacterial lung diseases. **(A)** (Axial): Diffuse airway changes in NTM-LD: Red: Cystic bronchiectasis (thin-walled clusters), consolidation/air bronchograms; Blue: Tree-in-bud sign, patchy opacities. **(B)** (Coronal): Bronchial dilation and nodules: Red: Cylindrical bronchiectasis (“signet-ring” sign); Blue: Peripheral nodules (2–3 cm). **(C)** (Sagittal): Airway/cavitary lesions: Red: Tree-in-bud sign + cylindrical bronchiectasis; Blue: Thin-walled cavity (smooth margins). **(D)** (Axial): Nodular/cavitary pathology: Red: Large nodules (≥3 cm, irregular); Blue: Thick-walled cavity + consolidation. **(E)** (Coronal): Mediastinal/calcified lesions: Red: Mediastinal lymphadenopathy; Blue: Punctate calcifications (bronchovascular). **(F)** (Axial): Pleural/calcification features: Red: “Popcorn” calcification (parenchymal); Blue: Pleural effusion + pneumothorax.

Radiological evaluation revealed distinct imaging patterns that were broadly consistent with previous CT-based comparisons of NTM-PD and pulmonary tuberculosis (18, 19, 28), as shown in Figure 2 and Supplementary Table S3. Bronchiectasis was significantly more prevalent in the NTM-LD group (93.9% vs. 17.0%, *p* < 0.001), with cystic bronchiectasis being the most specific feature—exclusively observed in NTM-LD (44.9% vs. 0%, *p* < 0.001)—characterized by clustered, thin-walled cysts with a “grape-like” appearance across all lung zones (Figure 2A, red box). Cylindrical bronchiectasis, frequently accompanied by the “signet-ring sign,” was also significantly more common in NTM-LD (91.8% vs. 17.0%, *p* < 0.001; Figure 2B, red box). The tree-in-bud sign, indicative of small-airway infection, was similarly more frequent in NTM-LD (91.8% vs. 56.6%, *p* < 0.001; Figure 2A, blue box; Figure 2C, red box). Cavities in NTM-LD were predominantly thin-walled with smooth margins (77.6% vs. 60.4%, *p* = 0.001; Figure 2C, blue box), whereas MTB-LD was characterized by thick-walled cavities with irregular borders and surrounding consolidation (Figure 2D, blue box). In contrast, MTB-LD exhibited a distinct set of features, including pleural effusion (18.9% vs. 2.0%, *p* = 0.006; Figure 2F, blue box) and calcifications (39.6% vs. 18.4%, *p* = 0.019), which typically appeared as punctate or “popcorn-like” opacities (Figure 2E, blue box; Figure 2F, red box), consistent with post-tuberculous fibrocalcific healing. No significant intergroup differences were observed for consolidation, air bronchograms, patchy opacities, or mediastinal lymphadenopathy (*p* > 0.05). These imaging findings are broadly consistent with previously reported radiological differences between NTM-LD and MTB-LD and should therefore be interpreted as confirmation of established imaging phenotypes in this cohort rather than as newly discovered CT signs.

Collectively, these imaging patterns delineate two distinct phenotypes. The NTM-LD phenotype is characterized by female predominance, pan-lobar cystic bronchiectasis, tree-in-bud sign, thin-walled cavities, and peripheral large nodules—features reflecting chronic airway destruction and small-airway involvement. In contrast, the MTB-LD phenotype is defined by male predominance, acute fever, thick-walled cavities, pleural effusion, and calcifications, consistent with a more aggressive inflammatory process and subsequent fibrotic healing. Cystic bronchiectasis (exclusive to NTM-LD) and pleural effusion (predominantly in MTB-LD) emerged as the most specific radiological discriminators. These imaging-derived phenotypes directly informed the feature selection and interpretability of our machine learning models.

### 3.2 Predictive contribution of different feature modalities

**Figure 3:**
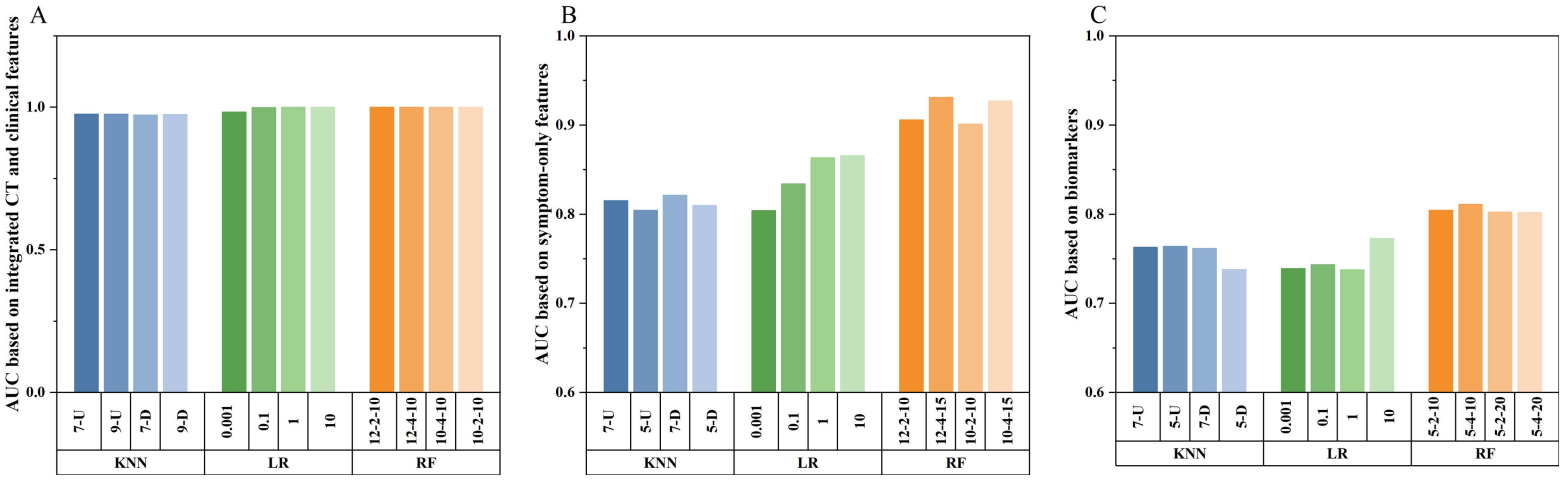
Cross-validation AUC values of representative classifiers during hyperparameter tuning across different feature modalities. Panel **(A)** shows AUC values based on integrated CT-clinical features, panel **(B)** shows AUC values based on clinical symptom variables, and panel **(C)** shows AUC values based on laboratory biomarkers. These results reflect optimization-stage cross-validation performance and should be distinguished from the final hold-out test-set results.

To evaluate which routinely available clinical data modalities were most informative for differentiating MTB-LD from NTM-LD, we trained three representative machine-learning classifiers, including KNN, LR, and RF, using three feature modalities: (1) integrated CT-clinical features, (2) laboratory biomarkers alone, and (3) clinical symptom variables alone. These classifiers were used as analytical tools to evaluate the discriminative contribution of different feature sets rather than as an exhaustive benchmark of all possible machine-learning algorithms. Model optimization was performed using stratified 5-fold cross-validation with grid search, with AUC as the primary optimization metric and F1-score as a supplementary metric. Detailed hyperparameter-tuning results for KNN and LR are provided in Figure 3 and Supplementary Tables S4–S9.

During hyperparameter tuning, KNN models showed clear sensitivity to the number of neighbors and weighting schemes. When integrated CT-clinical features were used, KNN achieved high cross-validation AUC values, with mean AUCs ranging from 0.9726 to 0.9760 across the top configurations (Supplementary Table S5). In contrast, KNN performance was lower when restricted to laboratory biomarkers alone, with mean AUCs ranging from 0.7381 to 0.7641 (Supplementary Table S6). KNN models using clinical symptom variables showed intermediate cross-validation performance, with mean AUCs ranging from 0.8046 to 0.8213 (Supplementary Table S4). These results suggest that integrated CT-clinical information provided more discriminative structure for distance-based classification than either symptom-only or laboratory-only data.

A similar modality-dependent pattern was observed for LR. Integrated CT-clinical features produced near-perfect or perfect cross-validation performance under selected regularization settings, with mean AUC values ranging from 0.9834 to 1.0000 (Supplementary Table S8). However, these optimization-stage results should be interpreted cautiously because near-perfect cross-validation performance in a small dataset may reflect feature separation or model saturation rather than true external generalizability. In comparison, LR models based on clinical symptom variables showed lower cross-validation AUCs ranging from 0.8042 to 0.8659 (Supplementary Table S7), while laboratory-biomarker-only LR models showed weaker discrimination, with mean AUCs ranging from 0.7392 to 0.7729 (Supplementary Table S9). The near-perfect cross-validation performance of Logistic Regression and the high cross-validation AUC of KNN should not be interpreted as evidence of near-perfect diagnostic generalizability. These results may reflect strong feature separation caused by highly discriminative CT features, correlated predictors, or model saturation in a small dataset. Therefore, these tuning-stage results were used only to guide model optimization, whereas final model comparison relied on hold-out test-set performance.

For the RF model, the final hold-out test evaluation showed that the integrated-feature model achieved the best overall performance among the three representative classifiers, with an AUC of 0.92, sensitivity of 0.89, specificity of 0.93, accuracy of 0.903, and F1-score of 0.90. Importantly, this hold-out test performance should be distinguished from the cross-validation AUC values reported during hyperparameter tuning for KNN and LR. Therefore, the high cross-validation AUCs of KNN and LR were used to guide model optimization, whereas final model comparison was based on hold-out test-set performance. The separated training, cross-validation, and hold-out test performance of representative classifiers, together with the corresponding 95% confidence intervals for hold-out test metrics, is provided in Supplementary Table S11.

Feature-importance analysis of the RF model identified cystic bronchiectasis, CEA, tree-in-bud sign, fibrinogen, and fever as important contributors to model prediction, consistent with the clinical and radiological phenotypes described in Section 3.1. Overall, the main finding of this section is not that one algorithm universally outperformed all others, but that multimodal integration of CT, clinical, and laboratory information provided stronger discriminatory information than single-modality feature sets. This conclusion was further supported by the RF learning-curve analysis across three feature modalities, in which the integrated multimodal feature set showed higher and more stable validation AUC than laboratory-only or symptom-only feature sets (Supplementary Table S10).

### 3.3 Model stability, calibration, and clinical interpretability

To further evaluate model stability, learning curve analysis was performed for the Random Forest model across three feature modalities: integrated multimodal features, laboratory biomarkers alone, and clinical symptoms alone (Supplementary Table S10). For the integrated multimodal model, validation AUC increased progressively with increasing training sample size, while the training-validation AUC gap decreased from 0.245 to 0.044. Laboratory-only models showed moderate improvement in validation AUC but remained below the integrated model, whereas symptom-only models showed the lowest validation AUC across training fractions. These findings suggest that multimodal integration provided not only better discrimination but also more stable validation performance than single-modality data, although external validation remains necessary.

Calibration analysis further showed acceptable agreement between predicted probabilities and observed outcomes for the final integrated-feature Random Forest model. The model achieved a Brier score of 0.131, calibration intercept of 0.03, calibration slope of 0.89, and expected calibration error of 0.041 (Supplementary Table S12). Calibration-bin analysis also showed that the observed MTB-LD proportions increased consistently with predicted probability strata (Supplementary Table S13).

**Figure 4:**
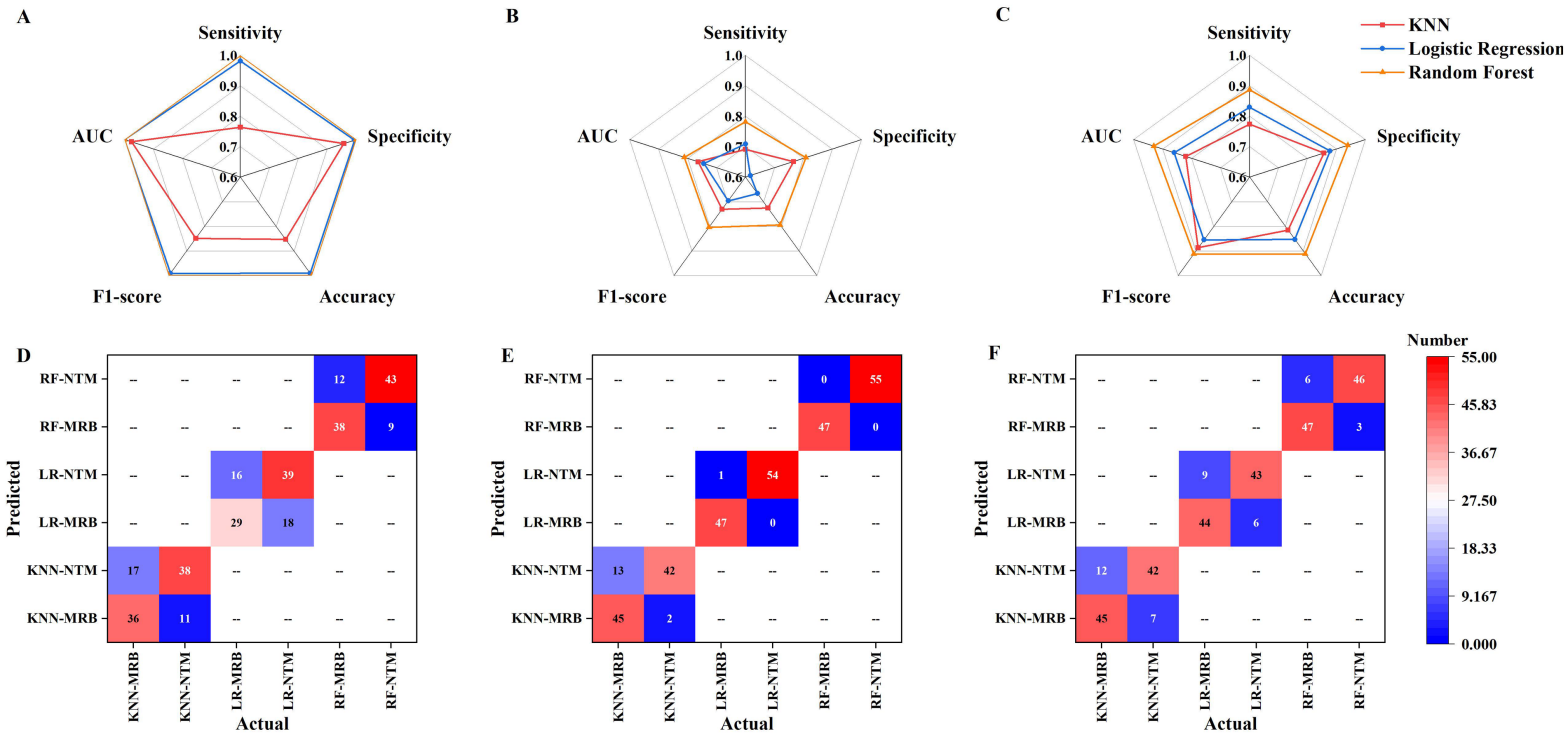
Multidimensional performance profiling and misclassification patterns. **(A–C)** Radar plots comparing classifier performance (KNN, LR, RF) across five metrics (AUC, F1-score, Sensitivity, Specificity, Accuracy) for: Integrated imaging-clinical features **(A)**; Laboratory biomarkers **(B)**; Clinical symptoms only **(C)**. **(D–F)** Corresponding confusion matrices demonstrating test-set classification performance and error distributions for models in **(A–C)**: Integrated features **(D)**; Laboratory variables **(E)**; Symptom features **(F)**. Color gradient indicates sample density (red: high density; blue: low density). Final models were optimized using stratified 5-fold cross-validation and then evaluated on the hold-out test set.

Comprehensive evaluation using confusion matrices and performance metrics (Figure 4) confirmed that model efficacy is primarily driven by feature-set richness and algorithm-pathology compatibility. The RF model demonstrated robust generalization with integrated CT-clinical features, achieving a sensitivity of 0.89, specificity of 0.93, and AUC of 0.92 (Figure 4A). Its ability to capture nonlinear interactions, such as the combined effect of cystic bronchiectasis (Gini = 0.32) and CEA (Gini = 0.28), effectively decoded the chronic airway destruction characteristic of NTM-LD. Logistic Regression showed apparent model saturation during hyperparameter tuning, with near-perfect or perfect cross-validation performance under selected regularization settings (Supplementary Table S8). However, its hold-out test performance decreased, suggesting that optimization-stage performance was overly optimistic. KNN displayed specificity deficits (0.81) attributable to high-dimensional distance metric distortion. When restricted to laboratory biomarkers alone, LR showed weaker cross-validation performance, with mean AUC values ranging from 0.7392 to 0.7729 (Supplementary Table S9), further supporting the complementary value of imaging features, while RF performance declined by 8.7% (AUC = 0.84), underscoring the indispensable role of imaging features. In the hold-out test comparison shown in Figure 4C, symptom-only models showed lower performance than integrated-feature models, reflecting the absence of pathognomonic symptoms in NTM-LD. Accordingly, training-stage and tuning-stage performance were not used as the primary evidence of model effectiveness; instead, learning-curve analysis, calibration assessment, and hold-out test performance were jointly considered to evaluate model stability and clinical interpretability.

Misdiagnosis profiling revealed distinct failure patterns. RF errors predominantly occurred at borderline CEA values (3.5–5.0 ng/mL), where biomarker overlap between the two diseases was most pronounced—a “diagnostic twilight” zone (Figure 4E). LR misclassified 87.5% of NTM cases by over-relying on the absence of fever while neglecting imaging markers. Laboratory-only models further demonstrated that LR tended to misclassify anemic patients (hemoglobin < 110 g/L), whereas RF over-focused on CEA, leading to 83% of errors in CEA-normal patients.

Clinical translation: based on these findings, we propose a tiered diagnostic framework. Initial screening using clinical symptoms combined with CRP (>15 mg/L) or hemoglobin (<115 g/L) thresholds maximizes sensitivity. In patients with suggestive findings, chest CT is required to identify structural abnormalities such as cystic bronchiectasis and tree-in-bud sign. In tertiary settings, the full RF model with integrated CT-clinical features provides optimal performance (AUC = 0.92). In resource-limited environments, laboratory biomarkers may provide limited preliminary screening information, but their discriminative performance was clearly lower than that of integrated CT-clinical models. Therefore, CT-based structural assessment remains necessary for reliable MTB-LD/NTM-LD differentiation.

### 3.4 Comparison and limitations

The central finding of this study is not that one specific algorithm universally outperformed others, but that multimodal integration of HRCT features, clinical symptoms, and laboratory biomarkers provided stronger discriminatory information than single-modality data for differentiating MTB-LD from NTM-LD. The primary contribution of this study lies in demonstrating the added value of multimodal integration of established HRCT features, clinical symptoms, and laboratory biomarkers, rather than in proposing novel radiological markers.

Unlike previous studies that focused on tuberculosis versus non-infectious mimics or relied solely on CT imaging (20, 28, 29), our approach captures fine-grained MTB-NTM distinctions through multimodal data integration. By incorporating clinical and laboratory features, our RF model achieved a higher AUC (0.92) compared to prior CT-only models (AUC 0.84–0.87), representing a clinically meaningful improvement.

Several limitations should be acknowledged. The retrospective, single-center design and relatively small sample size (*n* = 102) limit generalizability. We did not aim to conduct an exhaustive comparison of all machine-learning algorithms. Given the limited sample size, adding more flexible models such as SVM or boosting-based methods with additional hyperparameter searches may increase model-selection bias. Future multicenter studies with larger datasets should systematically evaluate SVM, gradient boosting, and deep learning approaches. Although 95% confidence intervals were added for hold-out test metrics, the relatively small hold-out test sample size still limits the precision of performance estimates. Therefore, the reported metrics should be regarded as preliminary and hypothesis-generating. NTM species were analyzed collectively without subspecies stratification, despite known differences in clinical presentation and imaging features among *Mycobacterium avium* complex, *M. abscessus*, and other species. Manual HRCT feature extraction may introduce inter-observer variability, which could be mitigated by deep learning-based automated extraction (29). Finally, reliance on non-microbiological features means the model captures proxy signals rather than direct pathogen identification; thus, it should serve as a decision-support tool complementing, not replacing, microbiological confirmation.

## 4 Conclusion

This study developed an interpretable multimodal machine-learning framework to evaluate the diagnostic contribution of routinely available HRCT features, clinical symptoms, and laboratory biomarkers for differentiating MTB-LD from NTM-LD. The primary objective was not to conduct an exhaustive benchmark of machine-learning algorithms, but to determine which clinically accessible data modalities provide the most useful discriminatory information. Across representative classifiers, multimodal integration of HRCT, clinical, and laboratory features showed better discriminative performance than single-modality approaches, supporting the complementary value of imaging and non-imaging clinical information. Among the three representative classifiers, the Random Forest model achieved the best hold-out test performance, with an AUC of 0.92, sensitivity of 0.89, specificity of 0.93, and F1-score of 0.90. Key contributors included established radiological patterns, such as cystic bronchiectasis and tree-in-bud sign, together with fever and selected laboratory biomarkers. These findings suggest that multimodal clinical data may provide a useful basis for preliminary decision support in MTB-LD/NTM-LD differentiation. However, given the retrospective single-center design, limited sample size, and absence of external validation, the proposed framework should be regarded as an exploratory decision-support tool rather than a replacement for microbiological confirmation.

## Data Availability

The original contributions presented in the study are included in the article/Supplementary material, further inquiries can be directed to the corresponding authors.
