# Supplementary material for: Precise discrimination of mycobacterial pulmonary diseases via multimodal machine learning integrating chest CT and clinical markers
Source: Front Med (Lausanne). 2026 Jul 9;13:1858126. doi: 10.3389/fmed.2026.1858126 (PMC13435664; doi:10.3389/fmed.2026.1858126)
Supplement: Supplementary file 1 [file Supplementary_file_1.docx]

**Supporting information**

**Precise Discrimination of Mycobacterial Pulmonary Diseases via Multimodal Machine Learning Integrating Chest CT and Clinical Markers**

Yangyi Jin^b, #^, Jindun Ding^a, #^, Jinsheng Ouyang^c^, Zhiye Yao^d, e^, Liping Wang^d. *^, Ruisong Xu^d, *^, Xuewen Jin^a, *^

^a^ Department of Respiratory and Critical Care Medicine, The People’s Hospital of Yuhuan, Yuhuan, Zhejiang, 317600, China

^b^ Department of General Internal Medicine, Zhejiang Cancer Hospital, Hangzhou, Zhejiang, 310022, China

^c^ Department of Respiratory and Critical Care Medicine, The First Affiliated Hospital of Wenzhou Medical University, Wenzhou, Zhejiang, 325000, China

^d^ Wenzhou Institute, University of Chinese Academy of Sciences, Wenzhou, 325001, Zhejiang, China

^e^ Terahertz Technology Innovation Research Institute, Terahertz Spectrum and Imaging Technology Cooperative Innovation Center, Shanghai Key Lab of Modern Optical System, University of Shanghai for Science and Technology, Shanghai, 200093, China

^#^ These authors contribute equally.

^*^ Corresponding E-mails: jxw045@163.com (X. J.); lpwang-wiucas@ucas.ac.cn (L. W.)

**Table S1.** Comparison of demographic characteristics and clinical symptoms between the MTB and NTM groups.

|  | **Characteristic** | **Participants with MTB (n=53)** | **Participants with NTM (n=49)** | **p value** |
| --- | --- | --- | --- | --- |
|  | **Age (years)** | 53 (24 to 84) | 49 (34 to 80) | 0.678 |
|  | **Sex** | / | / | <0.001 |
|  | Male | 34/53 | 14/49 | - |
|  | Female | 19/53 | 35/49 | - |
|  | **Place of Household Registration** |  |  | <0.001 |
|  | Within Province | 13/53 | 14/49 | - |
|  | Outside Province | 40/53 | 35/49 | - |
|  | **Place of Residence** |  |  | 0.644 |
|  | Urban Area | 13/53 | 14/49 | - |
|  | Rural Area | 40/53 | 35/49 | - |
|  | **Etiology** |  |  | <0.001 |
| MTB | | 53/53 | 0/49 | - |
| NTM | | 0/53 | 49/49 | - |
| **Clinical Symptoms** | |  |  | - |
| Dyspnea | | 2/53 | 6/49 | 0.112 |
| Chest Tightness | | 5/53 | 3/49 | 0.534 |
| Chest Pain | | 2/53 | 6/49 | 0.112 |
| Fever | | 21/53 | 1/49 | <0.001 |
| Hemoptysis | | 11/53 | 11/49 | 0.835 |
| Cough | | 44/53 | 39/49 | 0.657 |

**Table S2.** Comparison of Peripheral Blood Routine, Inflammation and Tumor Markers between MTB and NTM Groups.

|  | **Characteristic** | **Participants with MTB (n=53)** | **Participants with NTM (n=49)** | **p value** |
| --- | --- | --- | --- | --- |
|  | **Complete Blood Count, CBC** | / | / | / |
|  | White Blood Cell Count (10^9/L) | 6.8264±1.9852 | 6.3592±3.04288 | 0.157 |
|  | Neutrophils (*10^9/L) | 4.6547±1.7354 | 5.1122±6.5381 | 0.024 |
|  | Lymphocytes (*10^9/L) | 1.7321±2.4319 | 1.4959±0.4936 | 0.176 |
|  | Eosinophils (*10^9/L) | 0.0932±0.0789 | 0.2041±0.4922 | 0.077 |
|  | Hemoglobin (g/L) | 130.3000±16.6020 | 121.3500±13.3630 | 0.171 |
|  | Hematocrit (%) | 0.3973±0.0489 | 0.3734±0.0367 | 0.064 |
|  | Platelets Count (*10^9/L) | 268.9800±81.9690 | 240.0400±80.7470 | 0.606 |
|  | Neutrophil Percentage | 66.8340±10.845 | 62.9020±11.6824 | 0.472 |
|  | Lymphocyte Percentage | 22.0076±9.1363 | 26.5837±10.2100 | 0.506 |
|  | **Inflammatory Biomarkers** | / | / | / |
| Fibrinogen (g/L) | | 4.6362±1.7189 | 3.5192±1.1506 | 0.001 |
| D-Dimer (mg/L) | | 0.9765±1.7020 | 0.5184±0.6834 | 0.010 |
| C-Reactive Protein (mg/L) | | 34.8188±38.9774 | 18.0150±36.1604 | 0.626 |
| Procalcitonin (ng/mL) | | 0.0929±0.1278 | 0.0550±0.0444 | 0.035 |
| **Tumor Markers** | | / | / | / |
| Carcinoembryonic Antigen, CEA(MIULAg/L) | | 2.0760±1.4856 | 1.5330±0.9107 | 0.087 |

**Table S3.** Comparison of chest CT imaging features between MTB and NTM groups.

| **Characteristic** | **Participants with MTB (n=53)** | **Participants with NTM (n=49)** | **p value** |
| --- | --- | --- | --- |
| **CT Imaging Features** |  |  | - |
| Nodules | 48/53 | 46/49 | 0.534 |
| Consolidation | 40/53 | 33/49 | 0.363 |
| Patchy Opacities | 52/53 | 46/49 | 0.271 |
| Air Bronchogram | 22/53 | 22/49 | 0.730 |
| Bronchiectasis | 9/53 | 46/49 | <0.001 |
| Cylindrical Bronchiectasis | 9/53 | 45/49 | <0.001 |
| Cystic Bronchiectasis | 0/53 | 22/49 | <0.001 |
| Cavitation | 32/53 | 38/49 | 0.001 |
| Interstitial Changes | 1/53 | 0/49 | 0.334 |
| Pleural Effusion | 10/53 | 1/49 | 0.006 |
| Tree-in-Bud Sign | 30/53 | 45/49 | <0.001 |
| Calcification | 21/53 | 9/49 | 0.019 |
| Mediastinal Lymphadenopathy | 9/53 | 5/49 | 0.320 |

**Table S4.** Hyperparameter-Tuned k-Nearest Neighbor Classification Performance on Comprehensive Symptom Variables (Mean F1-Score & AUC).

| **n_neighbors** | **weights** | **mean F1-score** | **mean AUC** |
| --- | --- | --- | --- |
| **7** | **distance** | **0.8871** | **0.8213** |
| 7 | uniform | 0.8613 | 0.8153 |
| 5 | distance | 0.8842 | 0.81 |
| 5 | uniform | 0.8758 | 0.8046 |

**Table S5.** Hyperparameter-Tuned k-Nearest Neighbor Classification Performance on Integrated Chest CT Imaging and Clinical Symptom Features (Mean F1-Score & AUC).

| **n_neighbors** | **weights** | **mean F1-score** | **mean AUC** |
| --- | --- | --- | --- |
| 9 | uniform | 0.8485 | 0.9760 |
| 7 | uniform | 0.8428 | 0.9757 |
| 9 | distance | 0.8572 | 0.9726 |
| 7 | distance | 0.8772 | 0.9747 |

**Table S6.** Hyperparameter-Tuned k-Nearest Neighbor Classification Performance on Peripheral Blood Routine Indices, Inflammatory Markers, and Tumor Biomarkers (Mean F1-Score & AUC).

| **n_neighbors** | **weights** | **mean F1-score** | **mean AUC** |
| --- | --- | --- | --- |
| 7 | uniform | 0.7308 | 0.7632 |
| 5 | uniform | 0.7314 | 0.7641 |
| 7 | distance | 0.7240 | 0.7620 |
| 5 | distance | 0.6897 | 0.7381 |

**Table S7.** Hyperparameter-Tuned Regularized Logistic-Regression Classification Performance on Comprehensive Symptom Variables

| **C** | **mean F1-score** | **mean AUC** |
| --- | --- | --- |
| 0.001 | 0.7537 | 0.8042 |
| 0.1 | 0.7941 | 0.8343 |
| 1 | 0.8554 | 0.8634 |
| 10 | 0.8214 | 0.8659 |

**Table S8.** Hyperparameter-Tuned Regularized Logistic-Regression Classification Performance on Integrated Chest-CT Imaging and Clinical-Symptom Features.

| **C** | **mean F1-score** | **mean AUC** |
| --- | --- | --- |
| 0.001 | 0.9876 | 0.9834 |
| 0.1 | 0.9908 | 0.9996 |
| 1 | 1 | 1 |
| 10 | 1 | 1 |

**Table S9.** Hyperparameter-Tuned Regularized Logistic-Regression Classification Performance on Peripheral Blood, Inflammatory and Tumor-Marker Variables.

| **C** | **mean F1-score** | **mean AUC** |
| --- | --- | --- |
| 0.001 | 0.6865 | 0.7392 |
| 0.1 | 0.6964 | 0.7435 |
| 1 | 0.6959 | 0.7378 |
| 10 | 0.7101 | 0.7729 |

**Table S10.** Learning curves of RF models across three feature modalities.

A. RF + Integrated multimodal features

| **Training fraction** | **Training samples** | **Training AUC, mean ± SD** | **Validation AUC, mean ± SD** | **Gap** |
| --- | --- | --- | --- | --- |
| 20% | 16 | 1.000 ± 0.000 | 0.755 ± 0.121 | 0.245 |
| 40% | 33 | 0.993 ± 0.010 | 0.842 ± 0.082 | 0.151 |
| 60% | 49 | 0.982 ± 0.014 | 0.881 ± 0.061 | 0.101 |
| 80% | 65 | 0.970 ± 0.020 | 0.905 ± 0.045 | 0.065 |
| 100% | 82 | 0.962 ± 0.025 | 0.918 ± 0.034 | 0.044 |

B. RF + Laboratory biomarkers alone

| **Training fraction** | **Training samples** | **Training AUC, mean ± SD** | **Validation AUC, mean ± SD** | **Gap** |
| --- | --- | --- | --- | --- |
| 20% | 16 | 0.982 ± 0.026 | 0.642 ± 0.143 | 0.340 |
| 40% | 33 | 0.958 ± 0.031 | 0.701 ± 0.104 | 0.257 |
| 60% | 49 | 0.931 ± 0.038 | 0.753 ± 0.085 | 0.178 |
| 80% | 65 | 0.902 ± 0.046 | 0.801 ± 0.067 | 0.101 |
| 100% | 82 | 0.887 ± 0.049 | 0.836 ± 0.052 | 0.050 |

C. RF + Clinical symptoms alone

| **Training fraction** | **Training samples** | **Training AUC, mean ± SD** | **Validation AUC, mean ± SD** | **Gap** |
| --- | --- | --- | --- | --- |
| 20% | 16 | 0.901 ± 0.071 | 0.551 ± 0.139 | 0.350 |
| 40% | 33 | 0.852 ± 0.068 | 0.586 ± 0.116 | 0.266 |
| 60% | 49 | 0.812 ± 0.059 | 0.612 ± 0.096 | 0.200 |
| 80% | 65 | 0.761 ± 0.053 | 0.643 ± 0.078 | 0.118 |
| 100% | 82 | 0.724 ± 0.049 | 0.667 ± 0.065 | 0.057 |

**Table S11.** Training, cross-validation, and hold-out test performance of representative classifiers using integrated CT-clinical features, with 95% confidence intervals for hold-out test metrics.

| **Model** | **Training AUC** | **Cross-validation AUC** | **Hold-out test AUC, 95% CI** | **Sensitivity, 95% CI** | **Specificity, 95% CI** | **Accuracy, 95% CI** | **F1-score, 95% CI** |
| --- | --- | --- | --- | --- | --- | --- | --- |
| KNN | 0.991 | 0.976 | 0.880 (0.756–1.000) | 0.875 (0.640–0.962) | 0.810 (0.559–0.931) | 0.839 (0.674–0.929) | 0.848 (0.684–0.935) |
| Logistic Regression | 1.000 | 1.000 | 0.850 (0.712–0.988) | 0.780 (0.536–0.916) | 0.867 (0.622–0.963) | 0.839 (0.674–0.929) | 0.840 (0.675–0.930) |
| Random Forest | 0.985 | 0.918 | 0.920 (0.818–1.000) | 0.890 (0.657–0.972) | 0.930 (0.698–0.987) | 0.903 (0.751–0.966) | 0.900 (0.747–0.965) |

Values in parentheses indicate 95% confidence intervals estimated using stratified bootstrap resampling of the hold-out test set.

**Table S12.** Calibration metrics of the final integrated-feature Random Forest model.

| **Metric** | **Value** |
| --- | --- |
| Brier score | 0.131 |
| Calibration intercept | 0.03 |
| Calibration slope | 0.89 |
| Expected calibration error | 0.041 |

**Table S13.** Calibration-bin analysis of the final integrated-feature Random Forest model.

| **Predicted probability bin** | **Mean predicted probability** | **No. of patients** | **Observed MTB-LD cases** | **Observed fraction** |
| --- | --- | --- | --- | --- |
| 0.00–0.10 | 0.05 | 5 | 0 | 0.00 |
| 0.10–0.30 | 0.17 | 5 | 1 | 0.20 |
| 0.30–0.50 | 0.36 | 5 | 2 | 0.40 |
| 0.50–0.70 | 0.64 | 5 | 3 | 0.60 |
| 0.70–0.90 | 0.82 | 5 | 4 | 0.80 |
| 0.90–1.00 | 0.94 | 6 | 6 | 1.00 |

**Table S14** Reporting checklist based on TRIPOD+AI principles and relevant CLAIM items.

| **Reporting domain** | **Information reported in this study** | **Manuscript location** |
| --- | --- | --- |
| Title and abstract | Study design, target disease, data modality, and ML framework described | Abstract |
| Background and rationale | Clinical need for MTB-LD/NTM-LD differentiation described | Introduction |
| Study design | Retrospective single-center exploratory study reported | Section 2.1 |
| Participants | Inclusion and exclusion criteria reported | Section 2.1 |
| Outcome definition | MTB-LD and NTM-LD defined by microbiological confirmation | Section 2.1 |
| Predictors | Clinical symptoms, laboratory biomarkers, and HRCT features described | Section 2.2 |
| Imaging feature definition | HRCT signs defined using predefined radiological criteria | Section 2.2 |
| Preprocessing | Missing value imputation, standardization, and one-hot encoding described | Section 2.4.1 |
| Model development | KNN, LR, and RF construction described | Section 2.4.2 |
| Hyperparameter tuning | Grid search and stratified 5-fold cross-validation described | Section 2.4.2 |
| Performance evaluation | AUC, sensitivity, specificity, accuracy, and F1-score reported | Sections 2.4.3, 3.2, 3.3 |
| Overfitting assessment | Learning curve analysis reported | Section 2.4.4; Table S10 |
| Calibration | Calibration curve, Brier score, calibration intercept/slope, and ECE reported | Section 2.4.1; Tables S12–S13 |
| Interpretability | RF feature importance reported | Sections 3.2–3.3 |
| Limitations | Small sample size, lack of external validation, and inter-observer variability discussed | Section 3.4 |
| Clinical use | Exploratory decision-support role clarified; not a replacement for microbiological confirmation | Sections 3.4 and 4 |
